# From the Laboratory to the Classroom: The Potential of Functional Near-Infrared Spectroscopy in Educational Neuroscience

**DOI:** 10.3389/fpsyg.2018.01840

**Published:** 2018-10-11

**Authors:** Guilherme Brockington, Joana Bisol Balardin, Guilherme Augusto Zimeo Morais, Amanda Malheiros, Roberto Lent, Luciana Monteiro Moura, Joao R. Sato

**Affiliations:** ^1^Center for Natural and Human Sciences, Universidade Federal do ABC, Santo André, Brazil; ^2^National Network of Science for Education, Rio de Janeiro, Brazil; ^3^Instituto do Cérebro, Hospital Israelita Albert Einstein, São Paulo, Brazil; ^4^NIRx Medizintechnik GmbH, Berlin, Germany; ^5^Departamento de Ciências Exatas e da Terra, Universidade Federal de São Paulo, Diadema, Brazil; ^6^Institute of Biomedical Sciences, Federal University of Rio de Janeiro, Rio de Janeiro, Brazil; ^7^D'Or Institute of Research and Education, Rio de Janeiro, Brazil; ^8^Center of Mathematics Computing and Cognition, Universidade Federal do ABC, São Bernardo do Campo, Brazil

**Keywords:** education, educational neuroscience, fNIRS, functional near-infrared spectroscopy, hyperscanning, classroom, eye-tracking, pupilometer

## Abstract

Paralleling two decades of growth in the emergent field known as educational neuroscience is an increasing concern that educational practices and programs should be evidence-based, however, the idea that neuroscience could potentially influence education is controversial. One of the criticisms, regarding applications of the findings produced in this discipline, concerns the artificiality of neuroscientific experiments and the oversimplified nature of the tests used to investigate cognitive processes in educational contexts. The simulations may not account for all of the variables present in real classroom activities. In this study, we aim to get a step closer to the formation of data-supported classroom methodologies by employing functional near-infrared spectroscopy in various experimental paradigms. First, we present two hyperscanning scenarios designed to explore realistic interdisciplinary contexts, i.e., the classroom. In a third paradigm, we present a case study of a single student evaluated with functional near-infrared spectroscopy and mobile eye-tracking glasses. These three experiments are performed to provide proofs of concept for the application of functional near-infrared spectroscopy in scenarios that more closely resemble authentic classroom routines and daily activities. The goal of our study is to explore the potential of this technique in hopes that it offers insights in experimental design to investigate teaching-learning processes during teacher-student interactions.

## Introduction

Since the proposal by Stokes (1997) that initiatives in science should be taken when inspired by a medical issue so that both their theoretical and applied returns are maximized, health indicators have significantly improved throughout the world (World Health Organization, 2015). However, the same trend has not occurred in education, as demonstrated by the poor performance of most countries on educational indicators (OECD, 2016). Translational research inspired by educational themes could, therefore, be of high social benefit and, in fact, it is emerging as a growing field worldwide, known as educational neuroscience (Meltzoff et al., 2009; Sigman et al., 2014; Master et al., 2016).

While this discipline is growing and developing, there is a simultaneous push by educators and policymakers for new educational practices and programs to be evidence-based (Slavin, 2002, 2008; McMillan and Schumacher, 2006; Kvernbekk, 2015). Educational neuroscience is expected to provide elementary findings regarding brain-functioning during the teaching and learning process with the goal of creating innovative and technological approaches that improve educational practices (Goswami, 2004; Szucs and Goswami, 2007; Colvin, 2016). However, the idea that neuroscience could influence teaching practice in the classroom is controversial (Bruer, 1997; Bowers, 2016). There is skepticism amongst researchers about the real possibility of using laboratory-derived results obtained from the use of neuroimaging techniques, such as functional magnetic resonance imaging (fMRI) or electroencephalography (EEG), to influence pedagogy. One criticism that also divides educators has to do with applying to their classrooms the results of brain research that uses artificial experiments and overly simplified tests to investigate cognitive processes.

To reduce the distance between the laboratory and school environment, experiments should be designed to capture ongoing brain activity and physiological phenomena derived from brains that are engaged in interactive and realistic scenarios as educational proxies. As a result of this process, we aim to get a step closer to the formation of data-supported classroom methodologies by employing functional near-infrared spectroscopy (fNIRS) in different paradigms to understand the mechanisms involved in educational processes as teaching and learning activities. Recently, fNIRS emerged as a neuroimaging technique capable of revealing the activation of different brain areas during a variety of cognitive tasks. fNIRS is relatively robust to subjects' body movements (Balardin et al., 2017b) and is, therefore, appropriate for naturalistic experiments such as social interaction between adults (Cui et al., 2012; Babiloni and Astolfi, 2014), developmental studies with infants (Taga et al., 2003; Minagawa-Kawai et al., 2008) and continuous monitoring (Galderisi et al., 2016; Balardin et al., 2017a).

Regarding the technological component of the experiment, near-infrared light is employed to study changes in relative concentrations of HbO and HbR (oxygenated and deoxygenated hemoglobin, respectively). Wavelengths for fNIRS systems are typically chosen within 650–900 nm, and at least two specific wavelengths are necessary to detect changes in the relative concentrations of both HbR and HbO ([HbR] and [HbO]) (Delpy et al., 1988).

Typical fNIRS measurements are obtained by using pairs of light sources and detectors placed on the scalp. The light crosses the cranial wall, taking an elliptical path, until being received by the photodetector placed nearby. Typically, source-detector separations of 30 mm are employed to achieve a balance between signal-to-noise ratio and to provide enough depth to reach the most superficial layers of the cerebral cortex (Strangman et al., 2013). Thus, a limitation of fNIRS is that it is incapable of recording deeper areas of the brain. Identification of a neural response, representing the activity of a given brain area, is based on neurovascular coupling (Huneau et al., 2015) and is recognized as a hemodynamic response.

The positive attributes of fNIRS cause it to be an attractive option for naturalistic investigation, especially in hyperscanning studies (simultaneous multiperson acquisition) designed to understand brain activation during social paradigms (Cui et al., 2012), including teacher-student interaction (Battro et al., 2013). To illustrate potential fNIRS applications in educational contexts, we report three case studies based on different experimental setups: two hyperscanning studies designed to provide data on neural mechanisms underlying cognitive processes in realistic teacher-student interactions in a school environment across subjects, and a third one utilizing fNIRS with mobile eye tracking on a single student.

## Case studies

These studies were approved by the Universidade Federal do ABC-Ethics Committee. We obtained informed and written consent from all adult participants and from the parents/legal guardians of all non-adult participants that took part in Experiments 1, 2, and 3. Besides his parents' consent, the 10-year-old boy in Experiment 3 also provided his own verbal agreement to participate. Experiments were performed following relevant regulations and guidelines.

### Experiment 1–teacher-student interaction

We aimed to demonstrate the feasibility of monitoring a child interacting with her teacher using fNIRS. Both were female, ages 4 and 23, respectively. In order to facilitate the child's compliance with the fNIRS setup, she was told the experiment room would be part of a spaceship in which she was going to space as an astronaut to play a game while wearing a special helmet, just like the teacher (the fNIRS cap with optodes). In this game, the context is a “space race” in which two small pieces of colored plastic represented the child and the teacher. They need to move these plastic pieces in a pathway marked with numbers. They need to reach the finish line at the end of this path to finish the space race.

**Figure 1:**
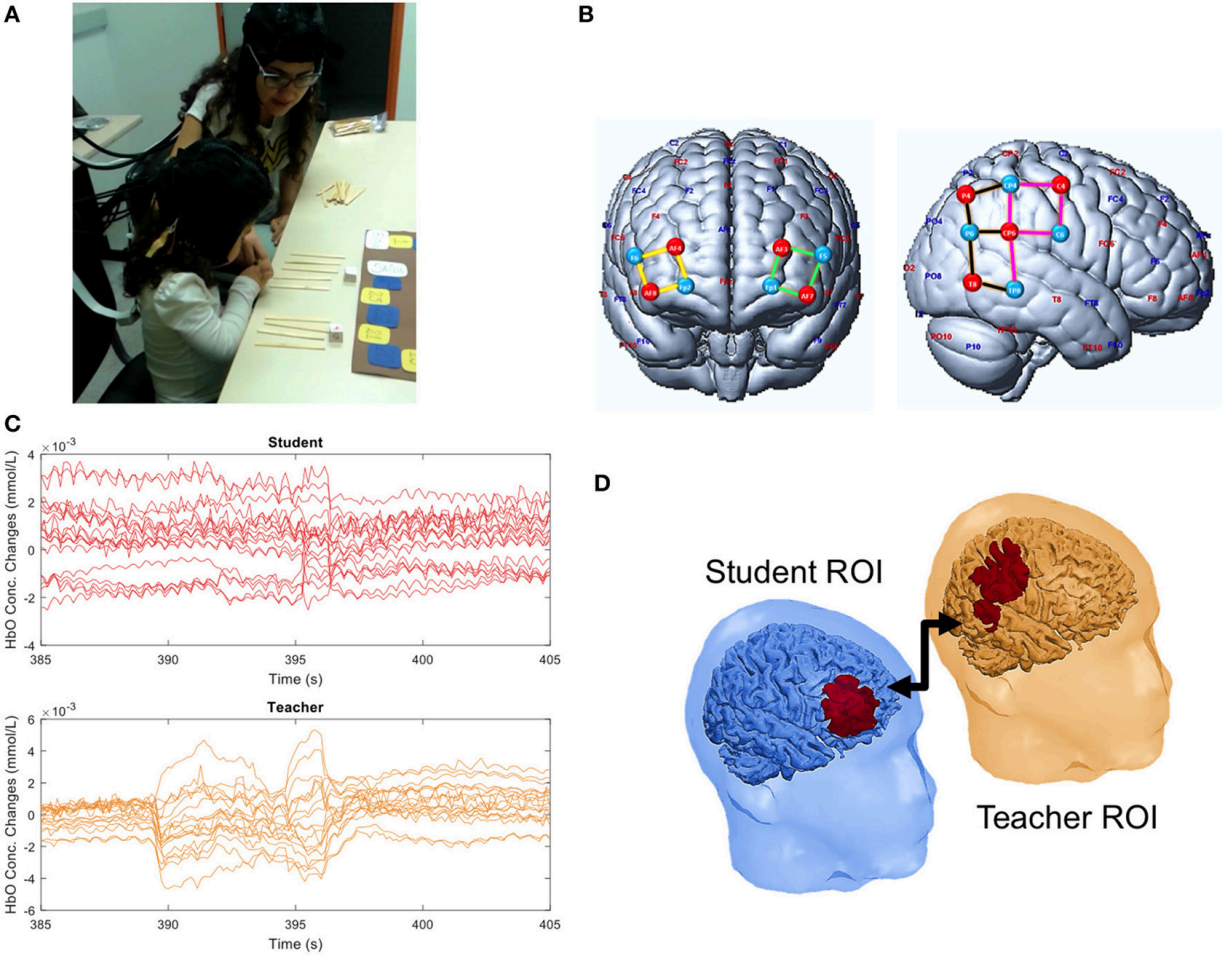
Experiment 1. **(A)** Teacher-student interaction during the counting board game. **(B)** Illustration of sources (red), detectors (blue), and channels grouped in four ROIs: left (green) and right (yellow) frontal cortex, anterior (pink) and posterior (brown) temporo-parietal junction. **(C)** Example of HbO concentration changes observed for each channel in the student and teacher during a time window of 20 s. **(D)** Significant student-teacher interbrain correlation. A written informed consent has been obtained from the depicted individuals (including the child parents) for the publication of this image.

The interaction task was a board game in which the teacher attempts to explain addition of two natural numbers (less or equal to six). This task was chosen considering the perspective of education as a cooperative social process occurring at the so-called proximal zone, as defined by Vygotsky (1998). First, the teacher verified that the child could count to twelve. Next, she briefly explained to her how to add two natural numbers using wooden sticks as tangibles. Two dice with six faces (numbered from 1 to 6) were tossed, and the two numbers had to be added together. The number of units to be run in the path was the sum of the two numbers. The child and the teacher alternated tossing the dice, but the addition operation was always carried out by the child with the help of the teacher (Figure 1A, Supplementary Video 1). Child-teacher interaction was continuously monitored with fNIRS.

The prefrontal cortex (PFC) is known to be involved in processes of high order cognition such as counting or calculating while the temporo-parietal junction (TPJ) is involved in social functions such as empathy and mentalizing (Fuster, 2000; Van Overwalle, 2009; Carter et al., 2012; Artemenko et al., 2018a,b; Soltanlou et al., 2018a,b). Thus, we hypothesized that activity in the teacher's TPJ would couple with that of the child's PFC. Therefore, we designed a cap montage with 18 channels focusing on these brain regions (Figure 1B). And the data acquisition was carried out using a NIRScout16x16 system (NIRx Medical Technologies, Glen Head, NY).

We found that the signal of the child's PFC was positively correlated with the teacher's anterior TPJ (correlation of 0.365, *p*-value = 0.010) (Figure 1D). Additionally, we found a correlation between the PFCs of the teacher and the child (correlation of 0.330, *p* = 0.016). These findings indicate the alignment of neural activity between child and teacher during a naturalistic educational interaction. Figure 1C depicts the raw HbO signals during a period of teacher-student interactions. Although it represents continuous speech and motion of both subjects, only a few artifacts can be visually identified. Thus, it supports the potential of fNIRS for this type of paradigm, even though further corrections may need to be applied.

### Experiment 2–group attention during a lecture

**Figure 2:**
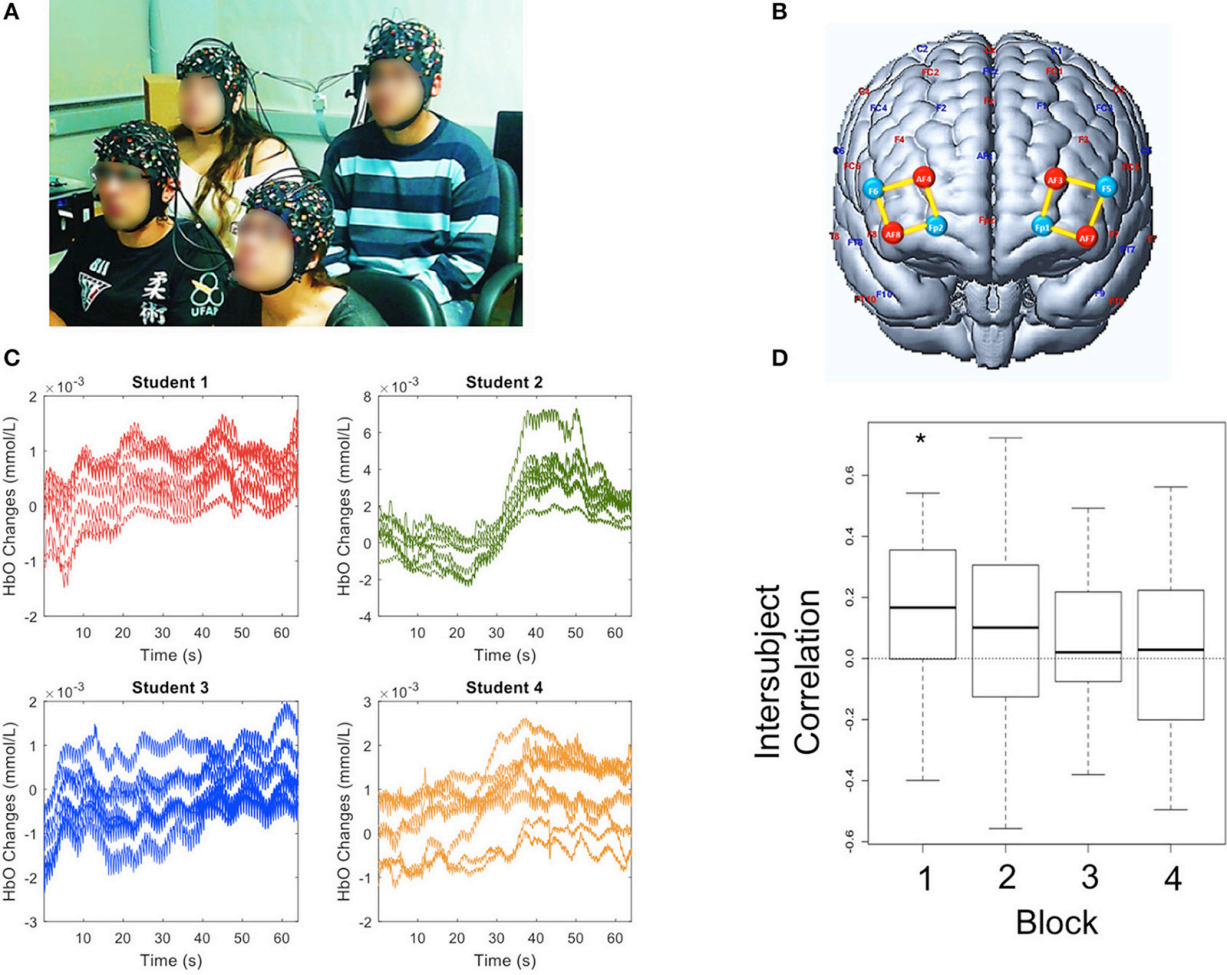
Experiment 2. **(A)** Group of students attending the class. **(B)** Illustration of sources (red), detectors (blue) and channels (yellow) over the bilateral prefrontal cortex. **(C)** Example of HbO concentration changes observed in each student's channels during a time window of 60 s. **(D)** Box-plots of inter-subject HbO correlations across four blocks of lecture. The horizontal line near the middle of each box indicates the median, while the top and bottom borders of the box mark the 25th and 75th percentiles, respectively. The asterisk (*) highlights a statistically significant difference from zero. A written informed consent has been obtained from the depicted individuals for the publication of this image.

Here, we provide a proof of concept of the feasibility of simultaneously monitoring hemodynamic signals of four undergraduate students (two of each gender) attending a lecture titled “Introduction to Epigenetics” (Figure 2A and Supplementary Video 2). Brain recording during the lecture was divided into four blocks lasting approximately 8 min each, with 2-min intervals. We hypothesized that the brain activity of both subjects would be coupled whenever similar attentional and arousal states were attained during the lecture. The optodes montage over the bilateral prefrontal cortex was the same for all subjects (Figure 2B).

The oxyhemoglobin signal (Figure 2C) was significantly synchronized amongst the subjects only during the first lecture block (Figure 2D: correlation of 0.17; *p* = 0.022). Interestingly, the interbrain coupling slightly decreased during the second block and was close to zero during third and fourth blocks (Figure 2D) indicating that a strong coupling effect between the students' brains resulted only during the first minutes of a lesson. This result may be related with students' broader attention span and/or higher arousal levels at the beginning which later decline.

### Experiment 3-simultaneous neurocognitive brain activity and overt attention during class

In this experiment, we explored the feasibility of simultaneously monitoring frontal fNIRS signals and the subject's gaze and pupil dynamics. Together, these three variables would associate overt attention to a limited region in space with changes in engagement, emotional response and cognitive effort of the student during the experiment (Võ et al., 2008; Wedel and Pieters, 2008).

One male child, ten years old, participated in this experiment. It consisted of simultaneous fNIRS and eye-tracking recording during a lecture titled “Introduction to Astronomy.” The teacher used a simple whiteboard for drawing pictures. Only the frontoparietal brain networks of the child were monitored since our primary targets were the attentional and cognitive control processes. The fNIRS cap montage consisted of 16 channels positioned bilaterally over frontal and parietal regions.

The experiment lasted 15 min during which the data acquisition was carried out using a continuous-wave fNIRS system (NIRSport8x8, NIRx Medical Technologies, Glen Head, NY, USA) with eight LED sources (760 and 850 nm) and eight detectors with resulting sampling rate of 7.91 Hz. The eye-tracker equipment was a monocular Mobile Eye-5 (ASL, Bedford, MA, USA) with a sampling rate of 30 Hz.

Results made possible a dynamic, descriptive account of concurrent hemodynamic, pupillometric and gaze signals during a realistic learning situation in late childhood. These findings may contribute to deciphering the efficacy of different teaching strategies during a lecture. As an example, Supplementary Video 3 highlights the importance of eye contact between student and teacher, even when the delivery of pedagogical information was written on a whiteboard.

## Discussion

In this Perspective article, we have demonstrated the feasibility of hyperscanning using fNIRS, along with other neurophysiological techniques, to record multiple brain activities and explore different paradigms of teaching and learning in academic contexts with three experiments.

In experiment 1, we found a positive correlation between the prefrontal cortex of a teacher and that of a child during an educational interaction which may be associated with the rule-following phase when monitoring of actions is critical for completion of a task (Cao et al., 2016). The most interesting finding, however, was the positive correlation between the teacher's temporal-parietal junction and the child's PFC. Learning is a bidirectional transfer of knowledge (Tovar-Moll and Lent, 2017) that relies on interpersonal negotiation during which the teacher initially serves as “strategic gatekeeper” and then dynamically changes to guide his behavior and the behavior of the student. The teacher's role is to anticipate the right moment and decide the manner in which he or she will deliver the information (LeBlanc and Bearison, 2004). In this context, the TPJ of the teacher plays a vital role given the involvement of this region in mental processing (Van Overwalle, 2009).

Regarding experiment 2, little is known about students' attention strategies, especially in naturalistic contexts. Attention is a frequent and crucial issue addressed by educators: “Since a student can learn only when she/he is in some way paying attention, a measure of learning time should include only time during which the student is engaged” (Fisher et al., 1981). However, how to address attention engagement of a student, especially in a group, is still a challenge in educational neuroscience. This case study was designed to measure the synchronicity of brain activity amongst four subjects attending a class. Sustained attention is an important component of scholastic achievement (Duncan et al., 2007; Steinmayr et al., 2010) and the PFC-basal forebrain system has a crucial role in mediating it. We hypothesized that time would be a crucial component in the students' ability to sustain attention (the shorter, the better). Our findings showed that intersubject PFC synchronization was strong only during the first quarter of a half-hour class, decreasing subsequently. Although many other factors may play a role, this result confirmed the hypothesis that teachers must help students sustain attention for a more extended period of time. These different variables deserve further research.

Although attention and engagement are amongst the most cited cognitive functions in education, how to objectively measure it is a great challenge. Here, we proposed a paradigm of investigation associating eye-tracking with fNIRS and demonstrated it to be feasible, as shown in experiment 3. As eye-tracker glasses serve the purpose of measuring attention from an outside perspective, it was especially interesting to observe how the student kept visual contact with the teacher while she was engaged in a Socratic-like dialogue with him. Simultaneously, it was possible to monitor brain activity and pupillary dilation which could be helpful to make inferences on arousal and attentional workload (Duchowski, 2007).

In conclusion, further investigations are necessary given the complexity of interpersonal interactions and the fact that education involves a bidirectional interaction between student and teacher (Tovar-Moll and Lent, 2017). Investigating the interface between neuroscience and education with more subjects and educational paradigms may provide data to support best practices in teaching and learning. This approach may provide new perspectives on the teaching-learning process through the discipline of educational neuroscience and, therefore, contribute to an evidence-based education.

## Ethics statement

All participants gave informed written consent in accordance with ethics approval by the Universidade Federal do ABC Ethics Committee, Brazil. The participants provided written consent to appear in the image published.

## Author contributions

GB and JS conceived and developed the experiments, discussed the results and wrote the manuscript. JS, JB and GZ executed the experiment, performed the computations, discussed the results and wrote the manuscript. AM, executed the experiments. RL verified the analytical methods, discussed the results and contributed to the final manuscript. LM discussed the results and wrote the manuscript.

### Conflict of interest statement

GZ was employed by NIRx Medizintechnik GmbH during study execution and manuscript preparation. The remaining authors declare that the research was conducted in the absence of any commercial or financial relationships that could be construed as a potential conflict of interest.

## Supplementary material

Supplementary Video 1 Example of HbO concentration changes (unfiltered signal) measured in the prefrontal and temporo-parietal regions of the student and the teacher during a time window of 20 s.

Supplementary Video 2 [HbO] time series of prefrontal cortex channels in each of the four subjects during an illustrative sample interval of 15 s of the lecture.

Supplementary Video 3 Illustration of student's fixation point (green circle) and pupil size (pink) as projected onto the screen during 20 s of the lecture.
